# Supplementary material for: Coexisting attractors in the context of cross-scale population dynamics: measles in London as a case study
Source: Proc Biol Sci. 2020 Apr 22;287(1925):20191510. doi: 10.1098/rspb.2019.1510 (PMC7211440; doi:10.1098/rspb.2019.1510)
Supplement: Supporting figures and information for data, modeling, and mixing analysis [file rspb20191510supp1.pdf]

Supplement to: Coexisting attractors in the context of cross-scale population dynamics: measles in London as a case study

SEIR model

The set of differential equations for the deterministic epidemic model are shown below. In practice, we confront the data with a stochastic version of this system where transitions are modeled as Euler-multinomial processes. Briefly, this process approximates the exit rate (e.g. due to infection or natural death) from a compartment over a small interval of time, Δt. For example, in the case of natural deaths (i.e., μ), the amount leaving the susceptible class, S, is approximated with size, S, and probability p = $\exp(-\mu\Delta t)$. More information can be found in (*1*)

$$\frac{dS}{dt} = Births - \lambda_t(I + \iota) - \mu S$$
$$\frac{dE}{dt} = \lambda_t(I + \iota) - \frac{1}{LP}E - \mu E$$
$$\frac{dI}{dt} = \frac{1}{LP}E - \frac{1}{IP}I - \mu I$$
$$\frac{dR}{dt} = \frac{1}{IP}I - \mu R$$

Here, *Births* are a known covariate from the data, ι is the mean infectious importation rate, μ is the death rate (where life expectancy is assumed to be 50 years), LP is the latent period (= 8 days), and IP is the infectious period (= 5 days). Further, force of infection can be modeled by:

$$\lambda_t = \beta(t) S N^{-1} W$$

with $\beta(t) = \overline{\beta}\,(1 + \alpha\sin(2\pi t + \phi))$, where $\overline{\beta}$ is the average transmission rate, α and ϕ the seasonal amplitude and phase. The transmissibility parameter, R_0, can be approximated as mean β_t/γ. Finally, W refers to a multiplicative white noise with standard deviation σ_{SE}. The case $\sigma_{SE} = 0$ refers to the fully deterministic model.

Calibration of the model with data

To fit the model to the observed weekly mortality data, *deaths*, we modelled true deaths (D) as the sum of CFRγI in a monthly period, where CFR is the inferred case fatality rate. We then model observed deaths, (i.e. *deaths*) via an over-dispersed binomial model per (*1*) with measurement error (e.g. false positives) (ψ). Although under-reporting could be estimated in this framework, we choose to fix it at 100% here as we assume mortality is well-reported.

$$P[deaths\,|D] = \Phi(deaths + 0.5; D, \psi^2 D^2) - \Phi(deaths - 0.5; D, \psi^2 D^2)$$

Where Φ refers to the cumulative normal distribution. When mortality is zero the likelihood is:

$$P[deaths\,|D] = \Phi(deaths + 0.5; D, \psi^2 D^2)$$

Using the above model and likelihood function, we then estimated parameters using Iterated filtering in the pomp R package (*3*, *4*). Briefly, this algorithm allows parameters (to be estimated) to take random walks in tandem, evaluating the likelihood at many parameter combinations. At each iteration, the standard deviation of the random walk is reduced, thus optimizing the likelihood of consecutive parameter sets. Once iterations are completed (set to be 60 for 400 unique parameter combinations), we have a set of parameters (N = 400), and their associated likelihood for each parameter combination. We choose the maximum likelihood estimate (MLE), and from here can forward simulate the fitted model (as per Figure 2).

Power spectrum analysis

For each of the nine regions and the city-level aggregate, we quantified periodicity through a power spectral analysis by taking the nearest-integer period corresponding to the maximum power. These results are shown below for the inner and outer regions. For each location, the dominant periodicity is the one with the largest power. Generally, clear periodicities can be observed for the aggregate and inner London. However, the Outer Ring appears to contain more noise, possibly due to their generally smaller size in comparison to the Inner London regions.

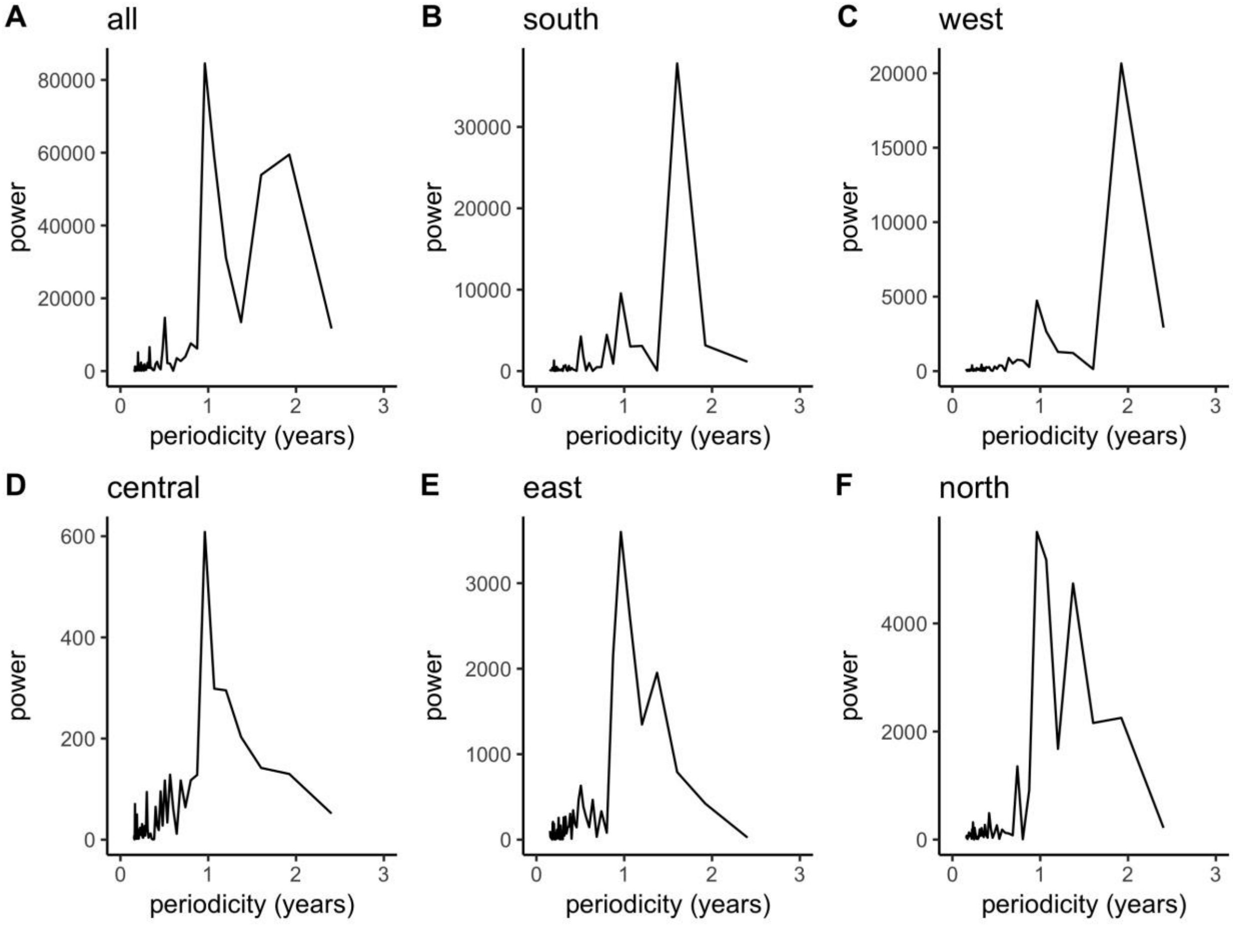

Figure S1: **Power spectrum of measles mortality for the five inner regions (plus the city-level aggregate) in London from 1897 to 1906.**

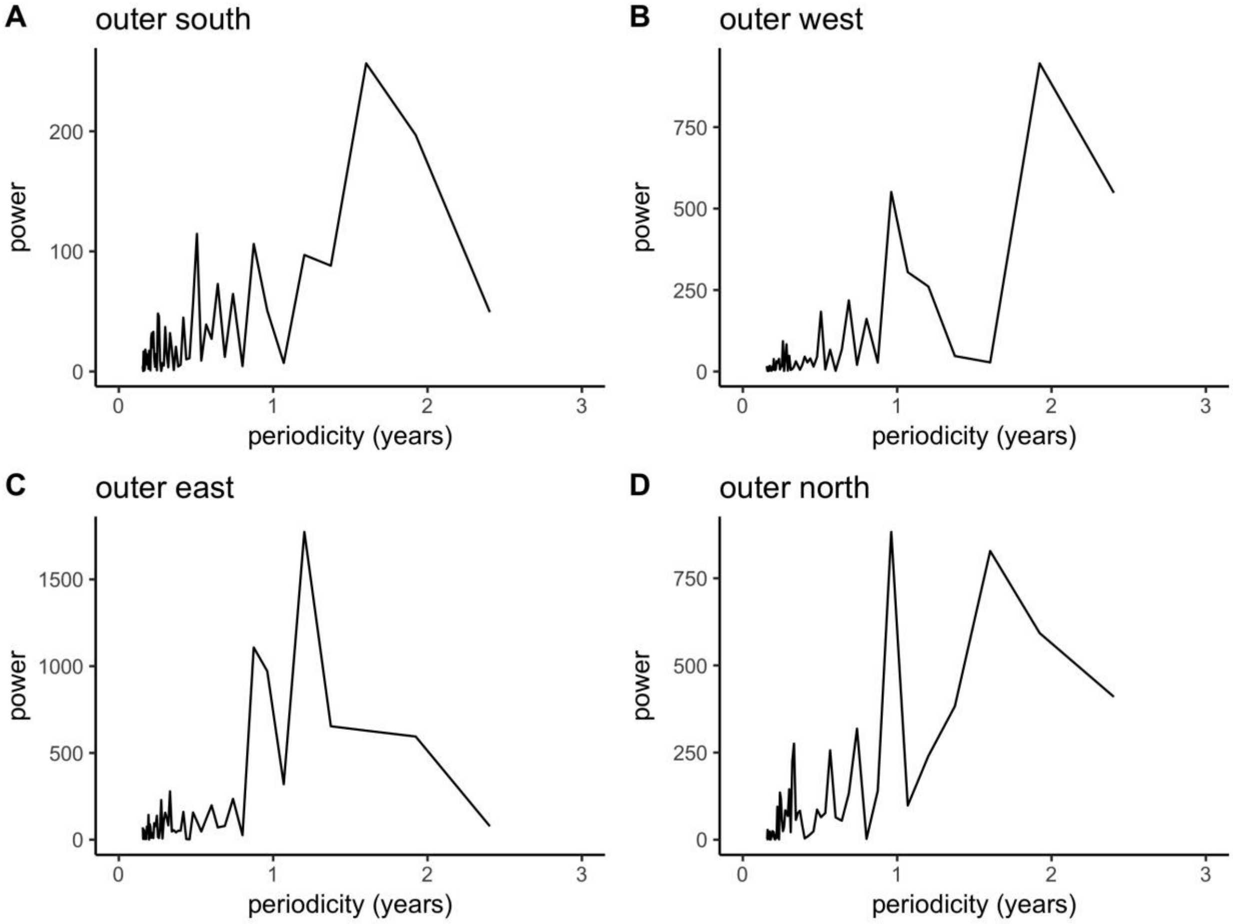

Figure S2: **Power spectrum of measles mortality for the four Outer Ring regions of London from 1897 to 1906.**

Outer ring model fits

Shown below are the 95% quantile model fits for the outer ring. These fits are significantly noisier than the inner London data, in particular outer South and outer West. However, we are still able to capture the larger 1898 outbreak, and the general epidemic amplitude for each region. The misfit in the outer South and outer West may point to variation in trajectories between stochastic simulations where not each simulation is biennial.

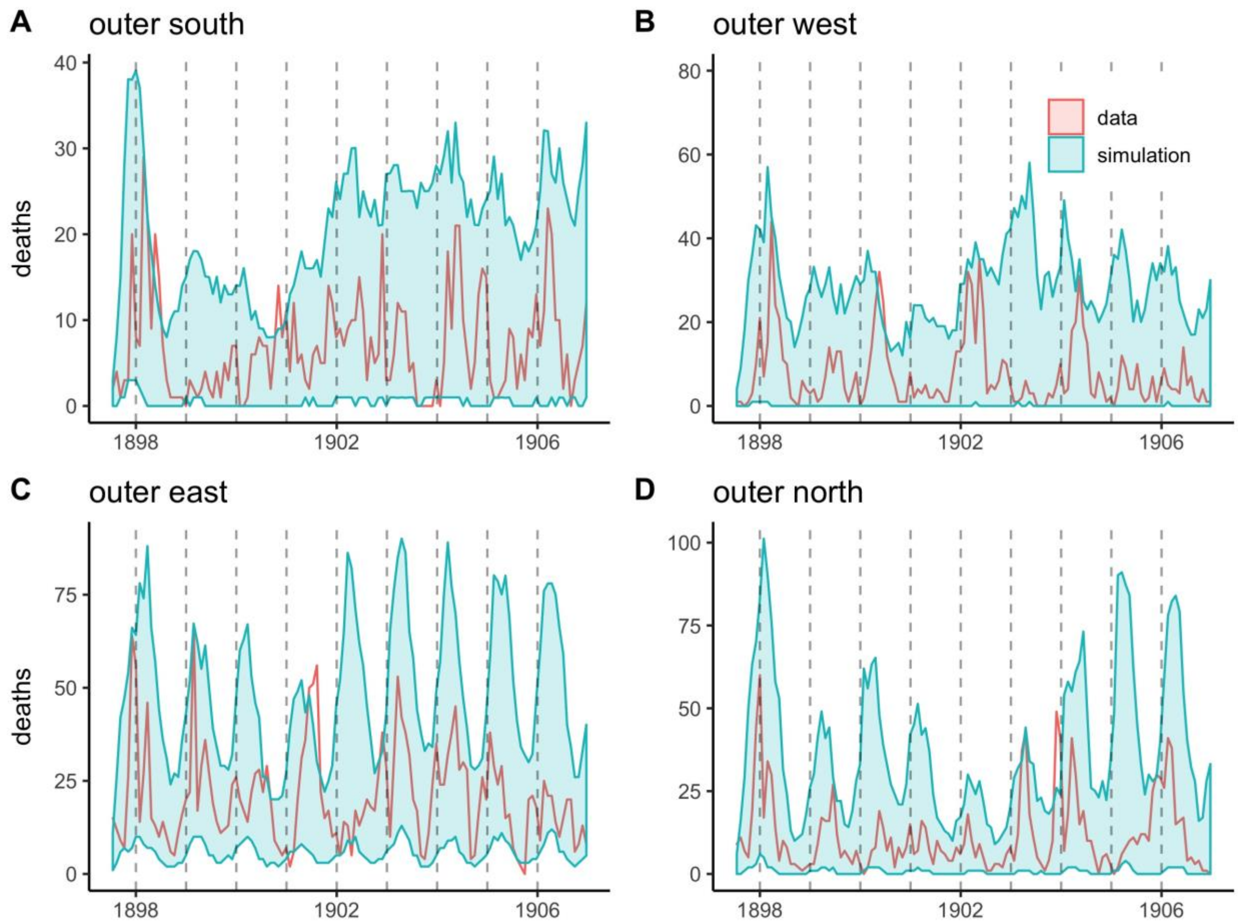

Figure S3: **Observed and predicted patterns of measles in outer London.** Simulating the fitted SEIR model yields generally close fits to the outer region-level data. The simulations, shown in light blue bands of 95% quantiles, visually match the observed data in terms of general amplitude, periodicity, and shape, even if slightly noisier. The fit to the South region indicates a potential mismatch to the data in terms of epidemic shape (discussed below).

Parameter estimates

In the table that follows, we show the estimated parameters for each location included in our analysis. Parameters refer to the above SEIR model description. Our inferred parameters broadly fell in line with previously analyses. We found a mean transmission rate, R_0, of 22, similar to the broad range described in (*2*, *5*, *6*). Initial conditions and multiplicative noise were similar to a later-era measles analysis (1950-1966) using the same framework (*2*). However, we did find a higher mean importation rate than previous analyses (again in the 1944-1966 era). Notably, we found a strong population dependence on the inferred importation rate (R^2 = 0.9, p-value < 1e-4), suggesting this may be a genuine feature of the data in this era.

	pop (x1000)	R_0	α	ϕ	σ_{SE}	ι	ψ	S_0	E_0	I_0	CFR
All	4,449	19	0.18	0.73	0.05	446.1	0.13	0.058	4e-05	6e-05	0.016
South	1,731	22	0.07	0.84	0.06	77.6	0.09	0.052	6e-05	6e-05	0.016

West	775	21	0.1	1.17	0.03	4.9	0.29	0.058	5e-05	5e-05	0.017
Central	273	25	0.18	0.34	0.1	24.3	0.08	0.046	4e-05	5e-05	0.017
East	681	21	0.16	0.49	0.06	93.1	0.24	0.056	6e-05	5e-05	0.017
North	987	21	0.19	0.70	0.06	53.2	0.16	0.057	6e-05	6e-05	0.015
Outer west	334	22	0.07	1.38	0.12	10.4	0.24	0.051	6e-05	5e-05	0.009
Outer east	579	20	0.17	0.45	0.08	79.0	0.09	0.049	5e-05	5e-05	0.013
Outer south	398	23	0.04	1.62	0.08	8.0	0.23	0.052	5e-05	5e-05	0.009
Outer north	475	22	0.19	0.75	0.12	22.5	0.06	0.059	6e-05	5e-05	0.01

Power spectrum fits

In addition to visual fits, we also evaluated the fitted model on power spectrum. We find generally close agreement with the data for this metric when we examine the period corresponding to the peak of the maximum power. However, the fit in the South region differed from the data, producing a mean bias around the annual attractor, although many simulations were still biennial when examining the 95% quantile (shaded band). Other regional fits, such as the West resulted in the correct periodicity, but a lower normalized power peak.

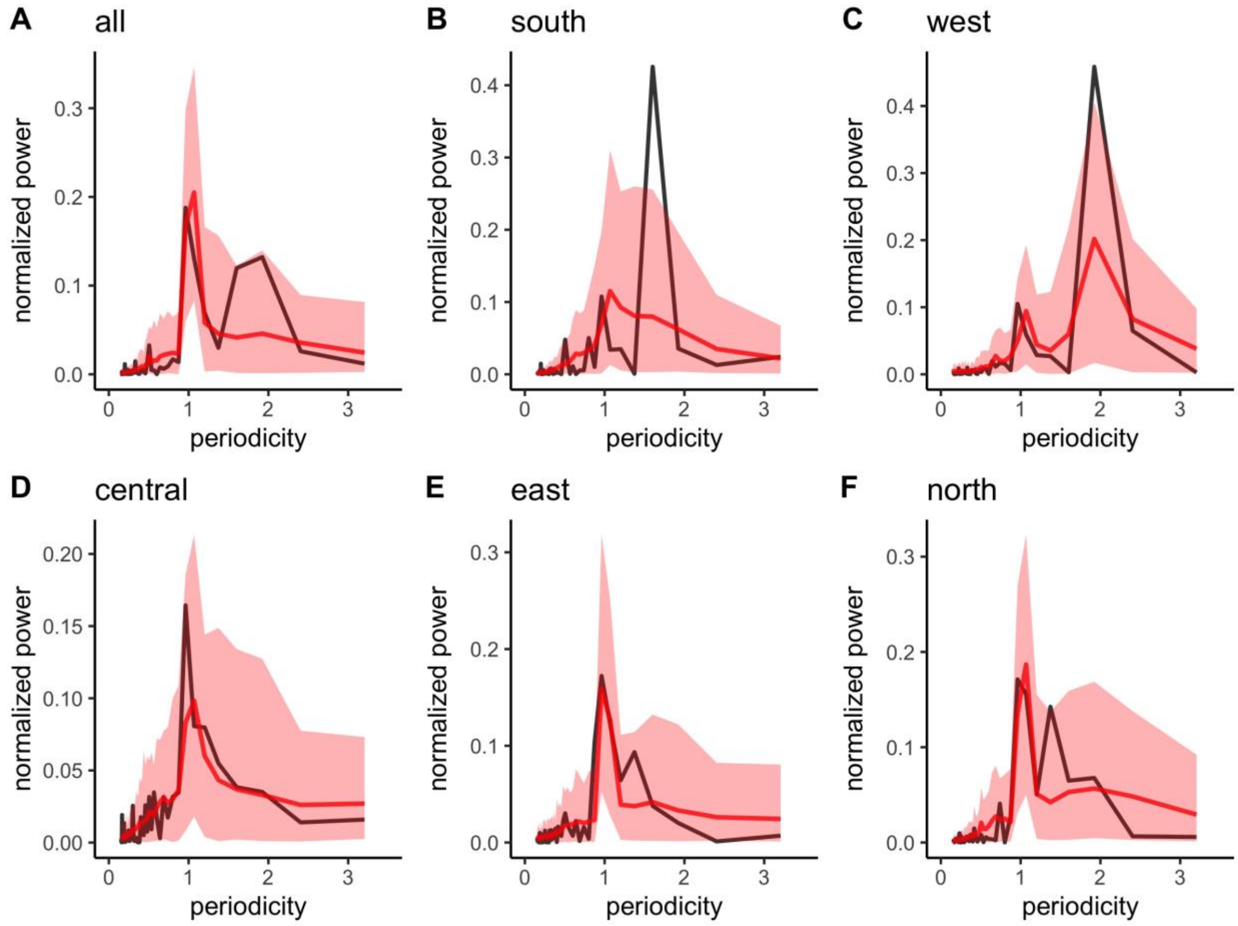

Figure S4: **Observed versus fitted power spectrum for inner London.** Simulating the fitted SEIR model (mean shown in red, 95% quantile bands shown in light red) yields generally close fits in terms of dominant frequency to the region-level data (black) when evaluated on power spectrum. We find a notable departure between the observed and simulated mean in the South

region indicating potential periodic mismatch when taking the simulation average. Additionally, when the simulated frequency is biennial, it fails to meet the same magnitude as the data.

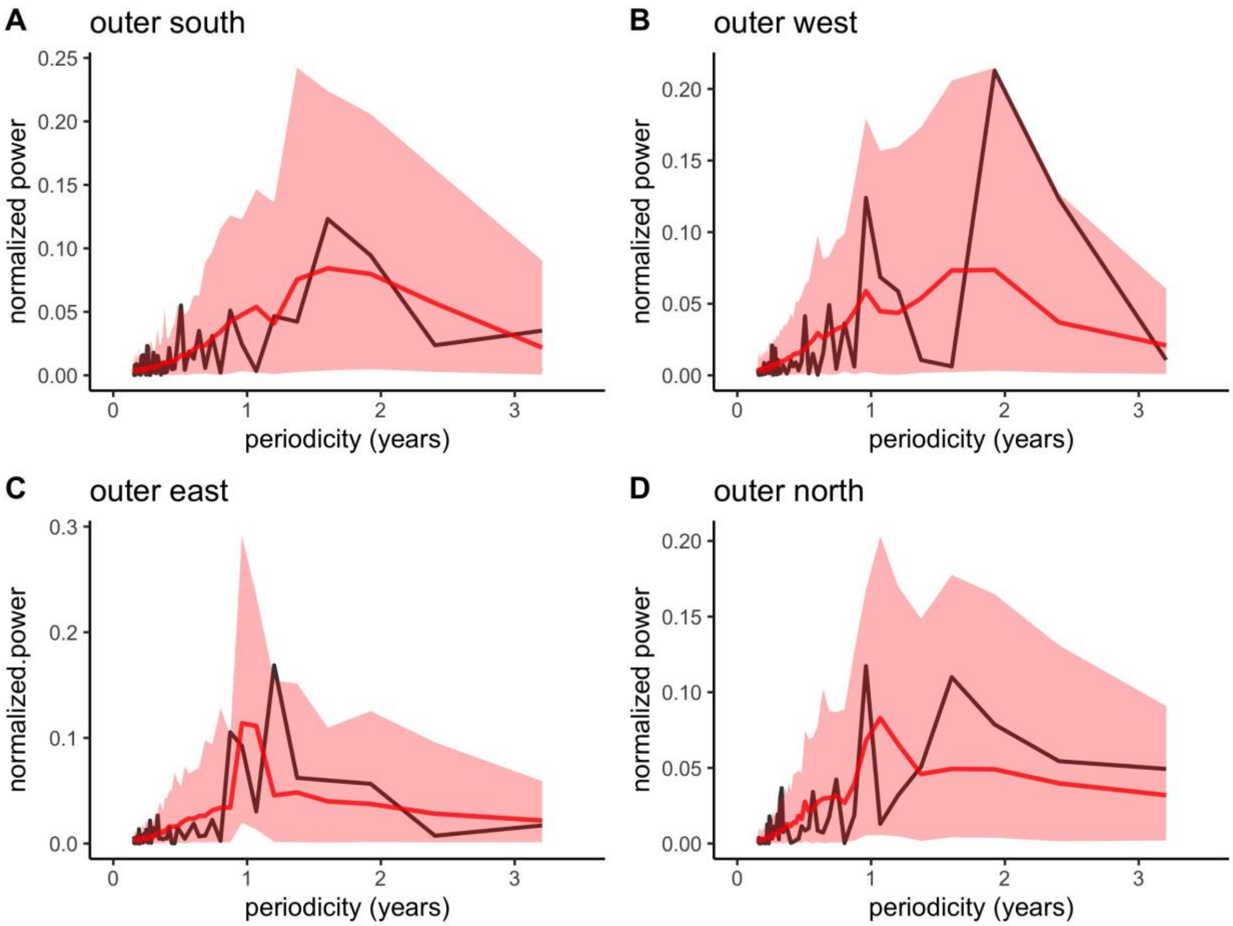

Figure S5: **Observed versus fitted power spectrum for outer London.** Simulating the fitted SEIR model (mean shown in red, 95% quantile bands shown in light red) yields generally the correct dominant periodicity when examining the peaks, although the mean simulations are much more dispersed as reflected through the large quantile bands.

East Region coupling

In addition to using the biennial West region as a case study for epidemic coupling, we also explored East London (annual dynamics). We find similarly strong agreement to the data, but notably a much larger range of potential coupling values for the East – outer East pairs, ranging from 0.09 – 0.47 (95% confidence interval). East and Central London also shared a much larger range of coupling than observed in the West region (95% CI: 0 - 0.26). These inferred parameters matched with the predicted dynamics, which showed strongly in phase dynamics, well. These results can be found in Figure S6.

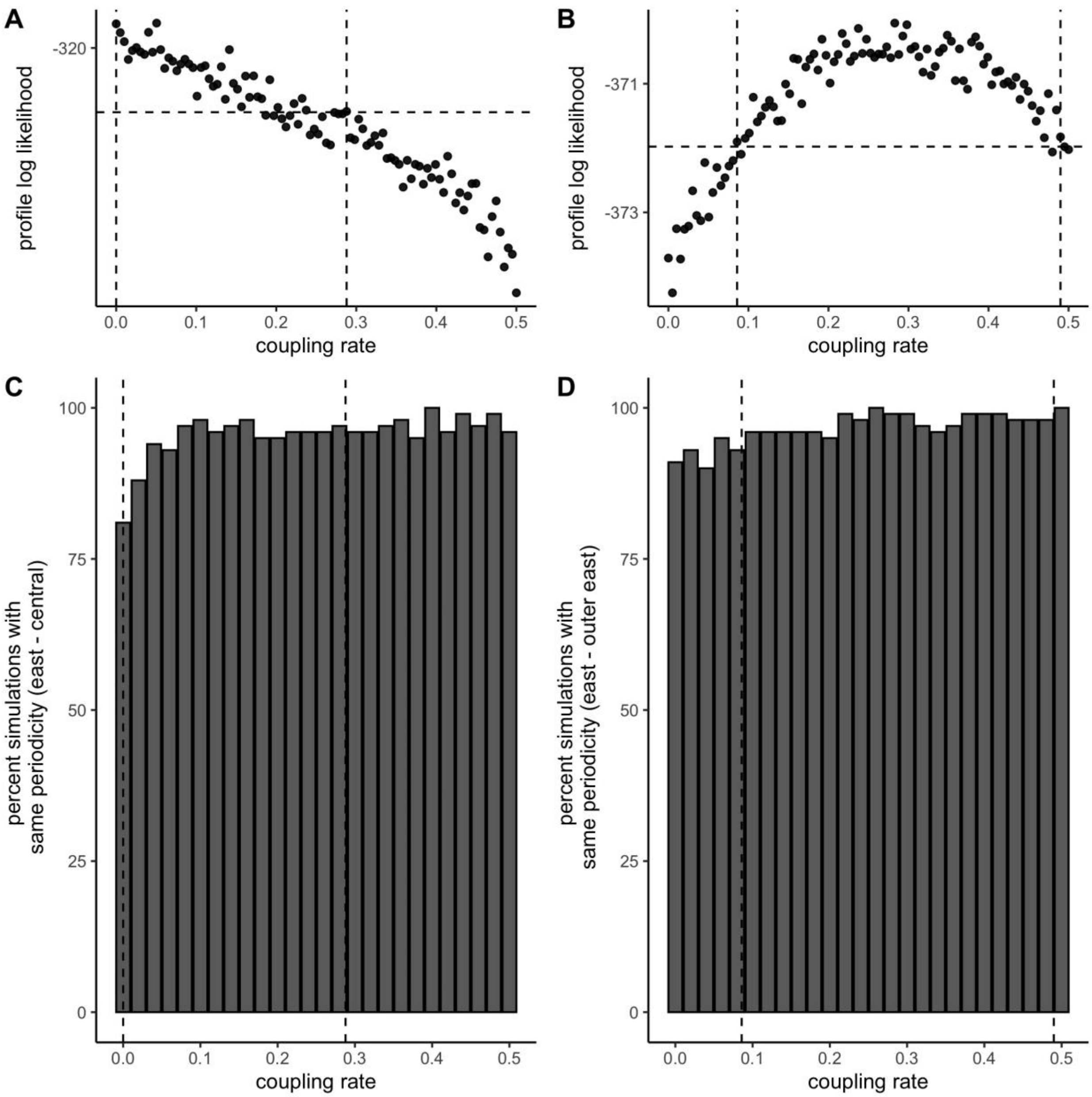

Figure S6: **The impact of spatial coupling on outbreak dynamics using East London as a case study. A.** The estimated coupling rate between the annual East and annual Central regions, where dashed lines indicate the 95% confidence intervals. **C.** The percent of simulations which resulted in outbreaks with the same periodicity for each coupling rate between East and Central London. The two dashed vertical lines refer to the estimated coupling rate inferred from the pairwise model, shown in **A**. **B**. and **D**. show the same analysis, but for the annual East and outer annual East regions.

Pairwise region coupling rates

For the fourteen regional pairs, we estimated a coupling rate. These values were generally low, with the highest rates typically occurring between an inner and outer region pair, as shown below. The error bars refer to 95% confidence intervals and the filled circle indicates the inferred

point estimate. Of the fourteen pairs, only four had mixing rates statistically distinct from zero, indicating that at this time sub-regions in London may have acted as relatively distinct populations. We found no significant relationship between importation and coupling rates.

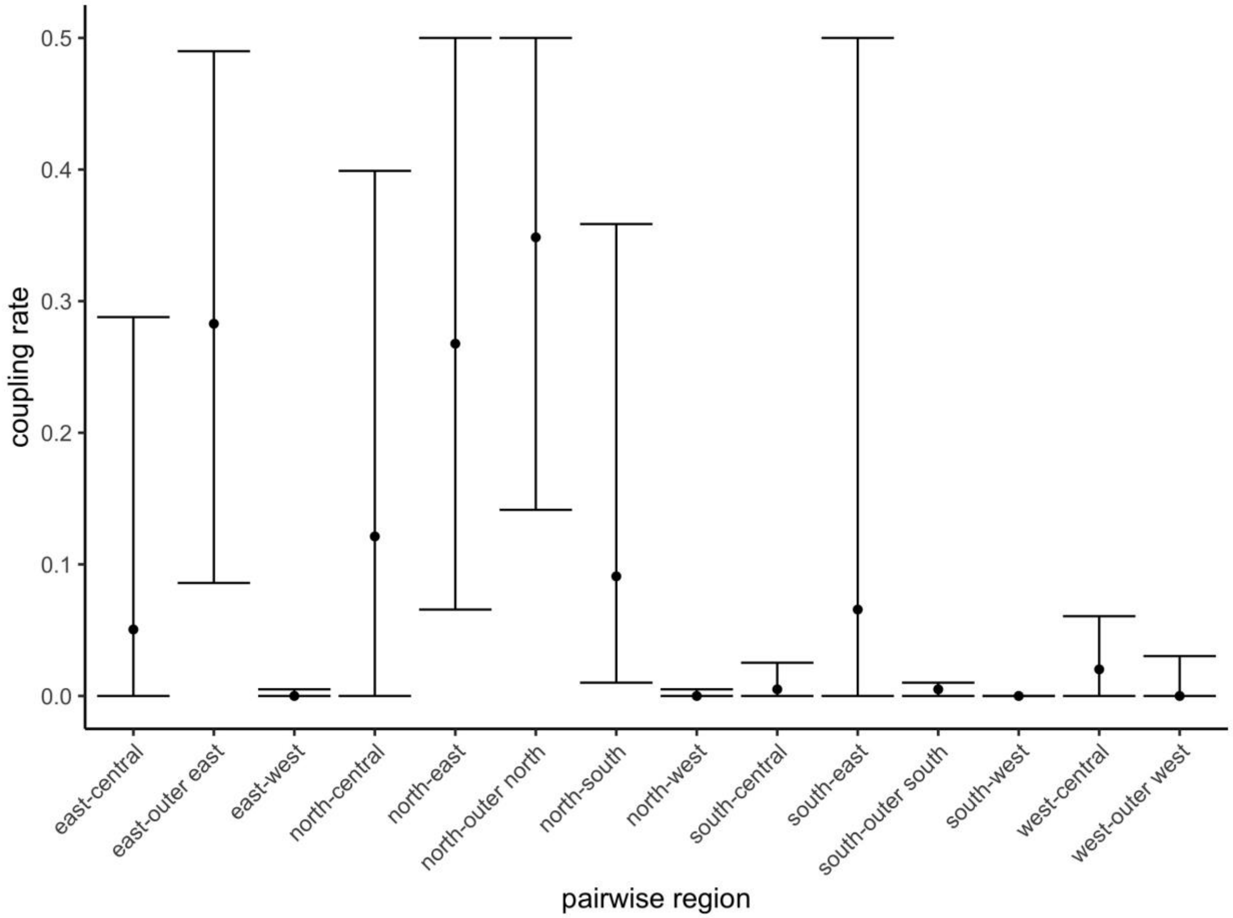

Figure S7: **The inferred coupling rates across region.** For each pairwise combination, we estimated a coupling rate. Here, the error bars refer to the 95% confidence interval, while the point refers to the estimate itself.

The role of coupling on periodicity

In order to explore the impact of various coupling rates on seasonality, we constructed a theoretical two patch model. We generated two populations, one consisting of 1,000,000 individuals (denoted P_1) and another of just 100,000 (denoted P_2). We explored the following two scenarios: when P_1 is annual and P_2 biennial, and when P_1 is biennial and P_2 annual. In order to fix the starting periodicities, we varied seasonality parameters as in Figure 3, using phase, $\phi = 1$, and amplitude $\alpha = 0.05$ to generate annual dynamics, and using phase, $\phi = 0.5$, and amplitude $\alpha = 0.125$ to produce biennial trajectories. For these combinations, we then varied coupling from zero to one and assessed the relative impact on periodicity, as shown in Figure S8. While the deterministic dynamics exhibited variation in periodicity, for both scenarios (Figure S8A shows when P_1 is annual, S8B shows when P_1 is biennial), dynamics of the same periodicity occurred very quickly, at just a modest rate of coupling = 0.01. It may be possible

that the stochasticity, or further variation in regional specific parameters or covariates may contribute to periodic asynchrony at coupling rates > 0.01.

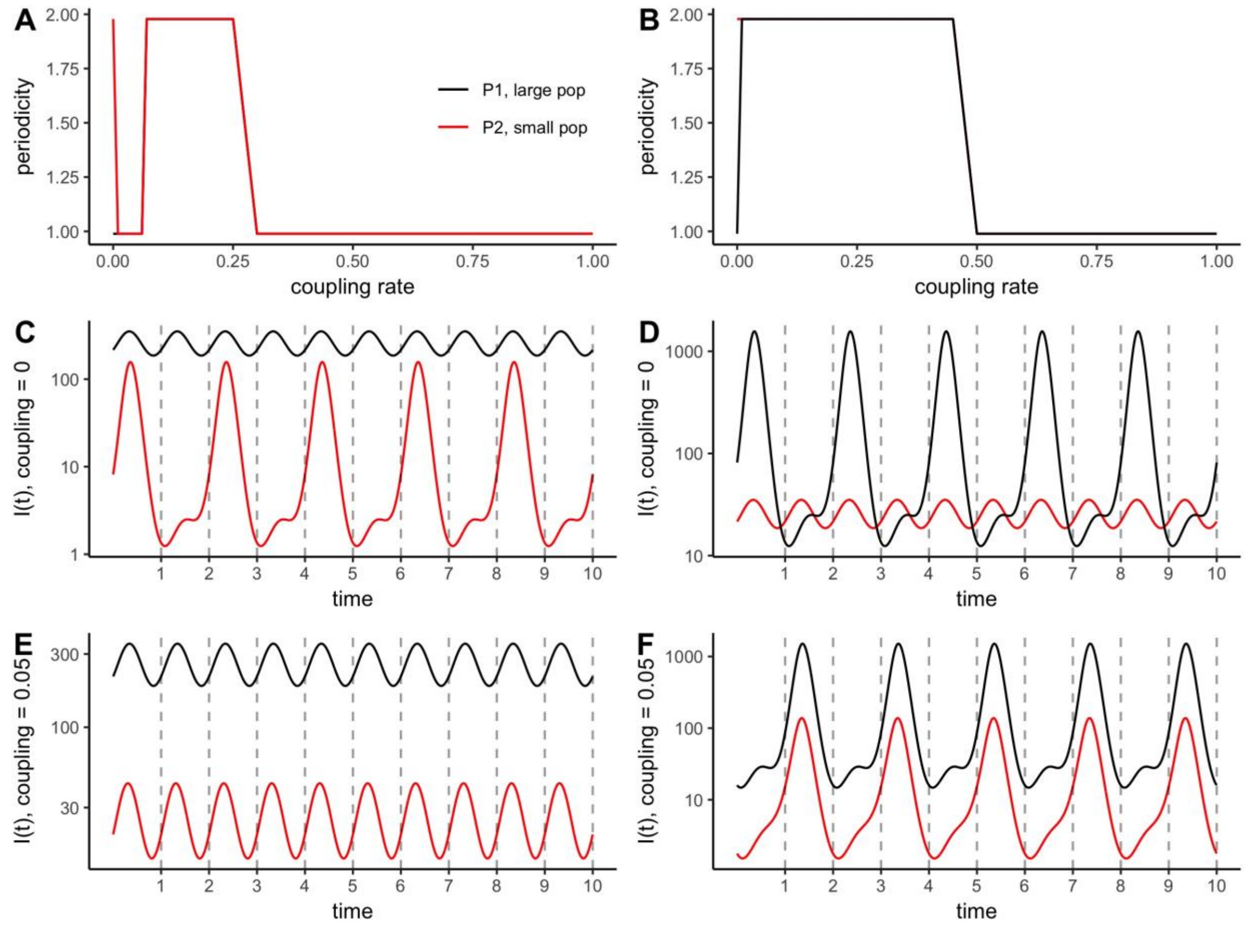

Figure S8: **The impact of coupling rate on producing dynamics of the same periodicty. A.** The impact of changing epidemic coupling when P_1 is biennial and **B.** the impact when P_1 is annual. Note in both situations, dynamics of the same periodic signal emerge rapidly at low values. Panels **C** and **D** show the respective time series for coupling rate = 0, where P_1 is initially annual (**C**) and biennial (**D**). In contrast, **E** and **F** the same situations, but for a coupling rate of 0.05. The dashed lines occur at every year.

Principal Component Analysis

To disentangle the role of birth rate and seasonality patterns such as phase and amplitude on periodicity, we performed a Principal Component Analysis (PCA). We found that 63% of the variation can be explained by PC1, of which amplitude and phase play the largest role when examining the loadings (0.69 and -0.65, respectively). Although birth rate is not unimportant (0.31) on PC1, the seasonal parameters yield a larger effect. Indeed, birth rate is more important in PC1 (0.94 versus -0.34 (amplitude) and 0.1 (phase)), however this axis, in contrast explains only less of the variation (31%).

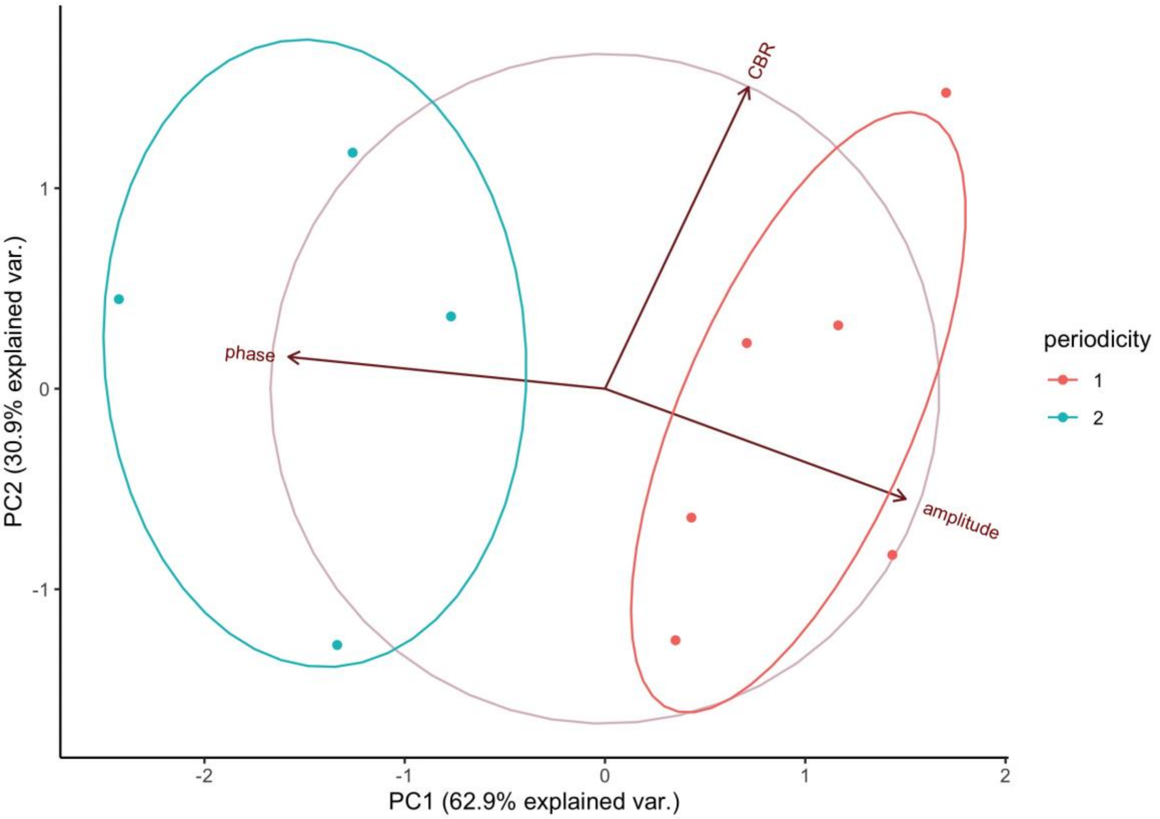

Figure S9: **Principal Component Analysis of seasonal phase, seasonal amplitude, and crude birth rate.** We find that although birth rate is not unimportant (PC1 loading = 0.31), both seasonal terms explain more variation (amplitude loading = 0.65, phase loading = -0.69).

Additionally, our fitted model estimated relatively large rates of outside importation across numerous regions. However, if we conduct a similar PCA analysis to above, seasonal amplitude and phase remain the primary drivers. However, future work on this dataset will examine the role of coupling in the context of a full spatially-explicit model.

Model convergence

To examine convergence of the iterated filtering algorithm, one can example traces for both the likelihood calculations as well as individual parameter combinations. Convergence has been achieved when the log likelihood has stopped increasing, as per Figure S10A. In the same way, parameters of interest, for example seasonal transmission as shown below, settle into their maximum likelihood estimate.

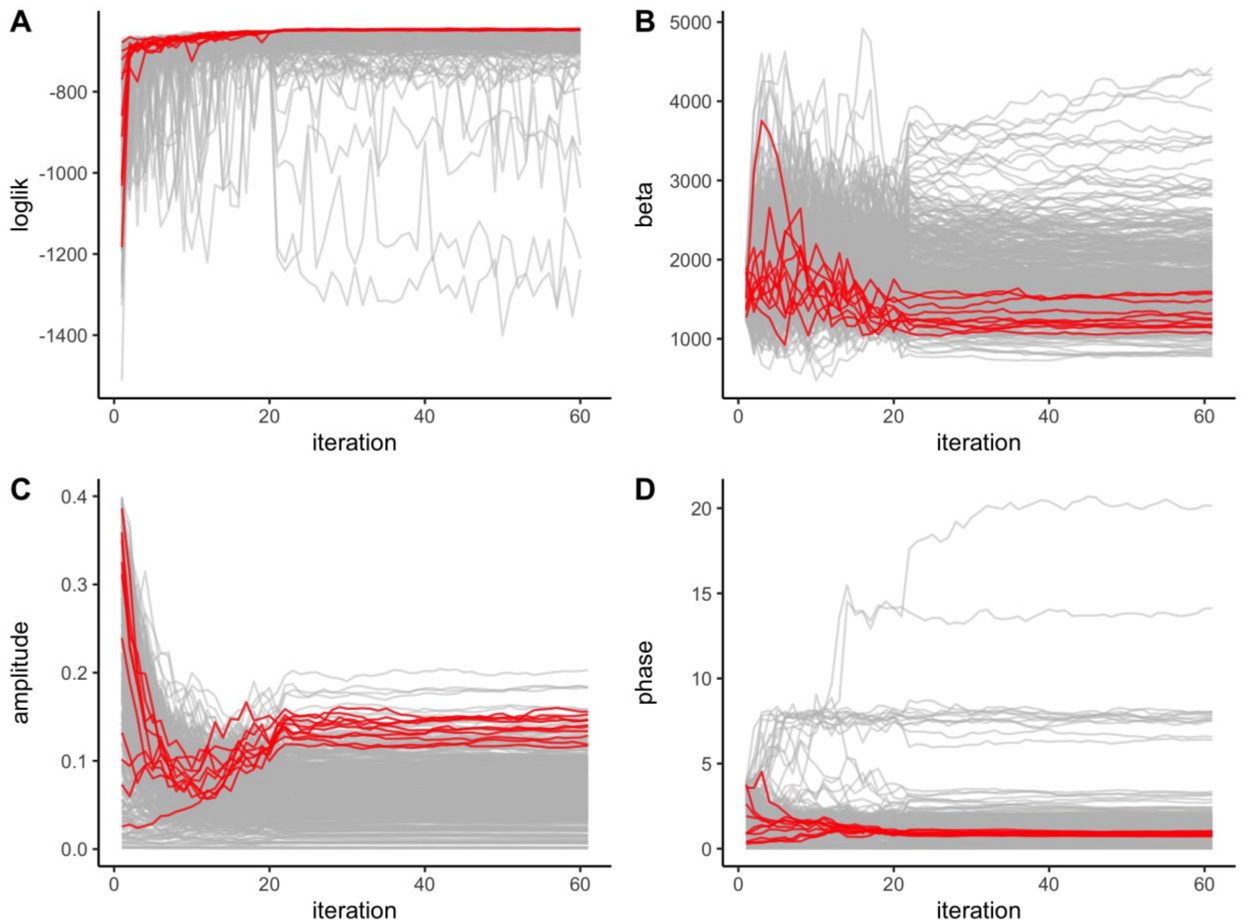

Figure S10: **Example of convergence using the iterated filtering algorithm for A) log likelihood and B-D) three seasonal transmission parameters.** Grey lines show the traces for each of the 400 parameter sets, with the red curves representing the best ten.

References:

1. Frequently asked questions, (available at https://kingaa.github.io/pomp/FAQ.html#eulermultinomial-approximation).
2. D. He, E. L. Ionides, A. A. King, Plug-and-play inference for disease dynamics: measles in large and small populations as a case study. *J. R. Soc. Interface*. **7**, 271–283 (2010).
3. A. A. King, D. Nguyen, E. L. Ionides, Statistical Inference for Partially Observed Markov Processes via the *R* Package **pomp**. *J. Stat. Softw.* **69**, 1–43 (2016).
4. A. A. King *et al.*, pomp: Statistical Inference for Partially Observed Markov Processes (2015), (available at https://cran.r-project.org/web/packages/pomp/index.html).
5. B. F. Finkenstädt, B. T. Grenfell, Time series modelling of childhood diseases: a dynamical systems approach. *J. R. Stat. Soc. Ser. C (Applied Stat.* **49**, 187–205 (2000).
6. B. D. Dalziel *et al.*, Persistent Chaos of Measles Epidemics in the Prevaccination United States Caused by a Small Change in Seasonal Transmission Patterns. *PLOS Comput. Biol.* **12**, e1004655 (2016).
